# Synthesis and Enhanced Light Photocatalytic Activity of Modulating Band BiOBr_X_I_1−X_ Nanosheets

**DOI:** 10.3390/nano11112940

**Published:** 2021-11-02

**Authors:** Bingke Zhang, Shengwen Fu, Dongbo Wang, Shujie Jiao, Zhi Zeng, Xiangyu Zhang, Zhikun Xu, Yaxin Liu, Chenchen Zhao, Jingwen Pan, Donghao Liu, Jinzhong Wang

**Affiliations:** 1 National Key Laboratory for Precision Hot Processing of Metals, Harbin Institute of Technology, Harbin 150001, China; zhangbingke007@163.com (B.Z.); wangdong165@sina.com (S.F.); zengzhi@hit.edu.cn (Z.Z.); zhangxiangyu@163.com (X.Z.); 20S009004@stu.hit.edu.cn (Y.L.); chenzhao@hit.edu.cn (C.Z.); Panjw19@163.com (J.P.); 19S009009@stu.hit.edu.cn (D.L.); 2 Department of Optoelectronic Information Science, School of Materials Science and Engineering, Harbin Institute of Technology, Harbin 150001, China; 3 College of Science, Guangdong University of Petrochemical Technology, Guandu Road No. 139, Maoming 525000, China

**Keywords:** BiOBr_X_I_1−X_, solid solution, photocatalytic degradation, band structure engineering

## Abstract

The photocatalysis technique has been proven to be a promising method to solve environmental pollution in situations of energy shortage, and has been intensively investigated in the field of pollutant degradation. In this work, a band structure-controlled solid solution of BiOBr_X_I_1−X_ (x = 0.00, 0.05, 0.10, 0.15, 0.20, 1.00) with highly efficient light-driven photocatalytic activities was successfully synthesized via simple solvothermal methods. The phase composition, crystal structure, morphology, internal molecular vibration, optical properties, and energy band structure were characterized and analyzed by XRD, SEM, HRTEM, XPS, Raman, and UV Vis DRS. To evaluate the photocatalytic activity of BiOBr_X_I_1−X_, rhodamine B was selected as an organic pollutant. In particular, BiOBr_0.15_I_0.85_ displayed significantly enhanced photocatalytic activity by virtue of modulating the energy band position, optimizing redox potentials, and accelerating carrier separation. Moreover, the enhancement mechanism was elucidated on the basis of band structure engineering, which provides ideas for the design of highly active photocatalysts for practical application in the fields of environmental issues and energy conservation.

## 1. Introduction

In recent years, solving energy shortages and environmental pollution has become a topic of great concern to the world [1,2,3,4]. Semiconductor photocatalysis technology is a green, low-cost technology that performs photocatalytic degradation of pollutants, photocatalytic hydrogen production, CO_2_ reduction, and other tasks [5,6,7]. However, semiconductor photocatalysts need to overcome disadvantages such as low light absorption rates, light quantum inefficiency, and high carrier recombination rates to meet the needs of further practical applications. Many efforts have focused on the exploration of high performance photocatalysts and the modification of visible-light-driven photocatalysts [8,9,10]. Some studies show that ion doping and substitution can optimize the crystal structure, adjust the bandgap, and effectively improve photocatalytic activity [11,12,13].

In addition to the advantages of stability and non-toxicity, ternary bismuth-based photocatalysts also have a unique energy band structure [14,15,16]. Unlike some metal oxide semiconductors composed of O 2p orbitals, the valence band of the Bi-based photocatalyst is formed by the hybridization of Bi 6s and O 2p orbitals [17,18]. The orbital hybridization reduces the forbidden bandwidth and disperses the valence band, thereby enhancing the visible light absorption capacity, making holes easy to move on the valence band, and hindering the recombination of photogenerated holes and photogenerated electrons [19,20,21]. BiOI has a narrow bandgap (1.7–1.9 eV), which can cover the entire visible light region, showing effective visible light photocatalytic activity. However, the narrow bandgap of BiOI leads to a high photogenerated electron-hole recombination rate. At the same time, the conduction band potential is lower than the O_2_/·O^2−^ reduction potential, and the valence band potential is higher than the OH^−^/·OH oxidation potential [22,23,24,25,26]. The adopted ion doping can overcome these unfavorable factors and further improve the visible light photocatalytic activity of BiOI [27,28]. Naturally, several preparation methods have been used to improve the structure and properties of materials, such as metal doping, non-metal doping, and solid solution [29,30,31,32]. Nevertheless, most studies have focused on the phenomenon that the decrease of the bandgap under ion doping leads to the increase of light absorption. In contrast, the effect of moderate widening of the bandgap on the photocatalytic performance of BiOI is rarely studied. It is worth noting that moderate widening of the band gap will reduce the effective absorption of light and the recombination efficiency of electron holes will be greatly reduced.

In this study, ion exchange was used to obtain the Br-substituted BiOI nanosheet photocatalysts (denoted as BiOBr_X_I_1−X_) by an easy and straightforward chemical precipitation method and to adjust the effect of energy band structure on the effective separation of electron holes and light absorption capacity. Various characterization methods were used to analyze BiOBr_X_I_1−X_ crystal structure, microscopic morphology, and other characteristics. The photostable organic degradation product rhodamine B (RhB) was selected to detect the photocatalytic activity of BiOBr_X_I_1__−X_, and the types of functional groups that play a key role in photocatalysis were determined by the quenching experiment of active groups. The moderate bandgap of BiOBr_X_I_1−X_ reduced the recombination probability of photogenerated carriers and adjusted the band potential, proving that the formation of BiOI solid solution significantly improved photocatalytic activity. It was noteworthy that the prepared BioBr_X_I_1-X_ material with a widened band gap changes the recombination probability of photogenerated carriers, which then affects the photocatalytic performance.

## 2. Materials and Methods

### 2.1. Synthesis of BiOBr_X_I_1−X_ Nanosheets

All chemicals were analytical grade drugs purchased from Aladdin (Shanghai, China) and were not further purified. First, 0.1455 g Bi (NO_3_)_3_.5H_2_O was dissolved fully in 30 mL glycol to form Solution A, and an appropriate amount of NaI and NaBr were dissolved in 30 mL deionized water to form Solution B. Then, Solution B was slowly added dropwise into Solution A, stirring the mixed solution from pale yellow to orange at room temperature until the color was no longer changing. Finally, the mixture was transferred to a teflon-lined stainless steel autoclave with a capacity of 100 mL for hydrothermal treatment at 120 °C/12 h. The resulting product was obtained by centrifugation, washed three times with water, and dried at 50 °C to obtain BiOBr_X_I_1−X_ (x = 0.00, 0.05, 0.10, 0.15, 0.20, 1.00) nanosheets.

### 2.2. Apparatus

Powder X-Ray diffraction (XRD) patterns (Empyrean, Panalytical, Malvern, UK) were used to characterize the crystal structure and phase composition. Raman spectra were measured on a laser micro-Raman spectrometer (LabRAM HR EV0, Horiba Jobin Yvon, Paris, France) using a 532 nm excitation laser. X-ray photoelectron spectroscopy (XPS) measurements were done with a spectrometer (ESCALAB 250Xi, Thermo Scientific Escalab, Waltham, MA, USA). The morphology of the materials was observed by field-emission scanning electron microscopy (SEM, Carl Zeiss, Merlin Compact, Jena, Germany), and TEM and HRTEM images were obtained on a Tecnai TEM G2 microscope (Waltham, MA, USA). The optical properties of samples were obtained from the UV-vis diffuse reflectance spectrum (Shimadzu UV1700, Shimane, Japan) and photoluminescence spectrum, with an excitation source at 532 nm wavelength.

### 2.3. Photocatalytic Activity and Photoelectrochemical Experiments

The light-induced photocatalytic performances of the as-prepared samples were measured with a RhB aqueous solution using a 300 W xenon lamp with a 400 nm cut-off filter and a light intensity of about 100 mW·cm^−2^. (Solar Light Company, Glenside, PA, USA). In a typical process, 15 mg of the sample was dispersed into a 30 mL RhB aqueous solution (10 mg/L). The suspension was stirred continuously in the dark for 30 min before illumination to achieve the sorption–desorption equilibrium. At regular 30 min intervals, the suspension system was sampled for analysis by a UV-visible spectrophotometer (Shimadzu UV1700). The electrochemical properties of materials were conducted on the electrochemical workstation. Under AM 1.5 simulated sunlight, the testing cell filled with 0.5 M Na_2_SO_4_ electrolytes comprised an Ag/AgCl electrode as the reference electrode, a Pt electrode as the counter electrode, and a conductive glass of tin fluoride oxide (FTO, OPV Tech, Yingkou City, China) coated with resulting samples as the working electrode.

## 3. Results

**Figure 1:**
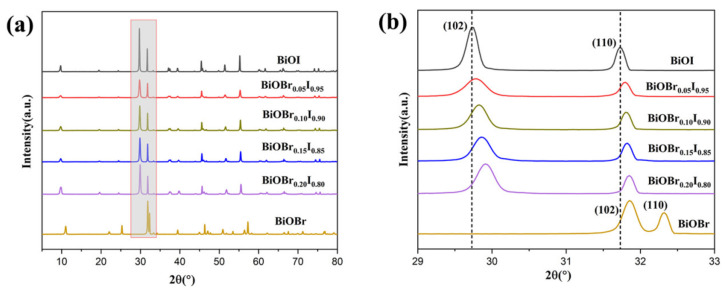
(**a**) XRD patterns of BiOBr_X_I_1−X_ samples (x = 0.00, 0.05, 0.10, 0.15, 0.20, 1.00). (**b**) The magnified portion of the patterns with 2θ ranging from 28.9 to 33.2°.

The crystallinity of synthesized photocatalysts were investigated using XRD. The XRD patterns of BiOBr_X_I_1−X_ samples are shown in Figure 1 and were found to be highly crystalline. There were two characteristic peaks at about 31.8 and 32.3°, which can be attributed to the (102) and (110) planes of BiOBr (JCPDS Card No.09-0393), respectively, and the major diffraction peaks that arrived at 29.7 and 31.7° are indexed to the (102) and (110) planes of tetragonal BiOI (JCPDS No.73-2062), respectively [33,34]. Remarkably, magnified XRD patterns of the peaks (110) and (200) (Figure 1b) showed that the diffraction peaks significantly shifted toward lower angles, the peak intensity gradually decreased, and the half height width widened with the increasing amount of Br, resulting from the generation of internal stress in the crystal after Br doping. These results demonstrated that the as-fabricated BiOBr_X_I_1−X_ samples were well crystallized solid solutions successfully doped with Br.

In order to further explore Br substitution doping into BiOI lattice, the Rietvled refined quantitative analysis was carried out. The samples’ lattice constants are shown in Table 1. It can be seen that the confidence factors Rwp and Rp of all samples were less than 15%, which proved that the refined results were reasonable. Due to the smaller radius of Br atoms than I atoms, the cell constant decreased with the increase of Br doping, indicating that Br-substitution replaces I in BiOI. Meanwhile, the average grain sizes of BiOBr_X_I_1−X_ (x = 0.00, 0.05, 0.10, 0.15, 0.20 and 1), calculated by Scheler formula, were 44.617, 30.832, 33.932, 33.330, 31.622, and 28.128 nm, respectively. It was concluded that after doping with Br, the average grain size of BiOI generally showed a downward trend, which is consistent with the previous analysis.

**Figure 2:**
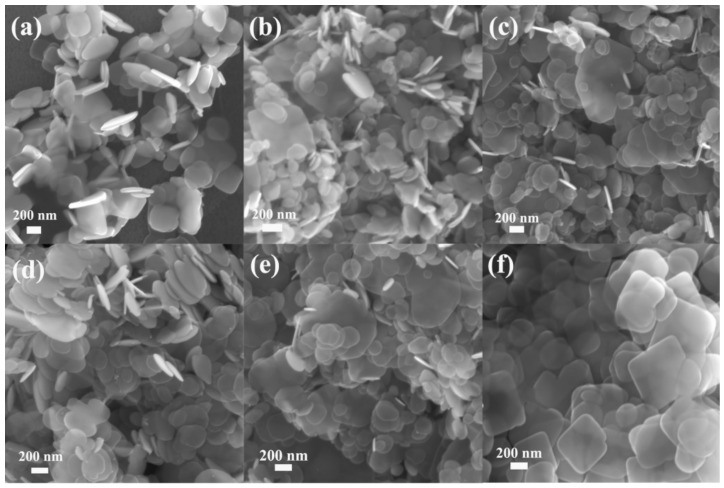
SEM image of ultrathin BiOBr_X_I_1−X_ nanosheets: (**a**) BiOI; (**b**) BiOBr_0.05_I_0.95_; (**c**) Bi-OBr_0.10_I_0.90_; (**d**) BiOBr_0.15_I_0.85_; (**e**) BiOBr_0.20_I_0.80_; and (**f**) BiOBr.

**Figure 3:**
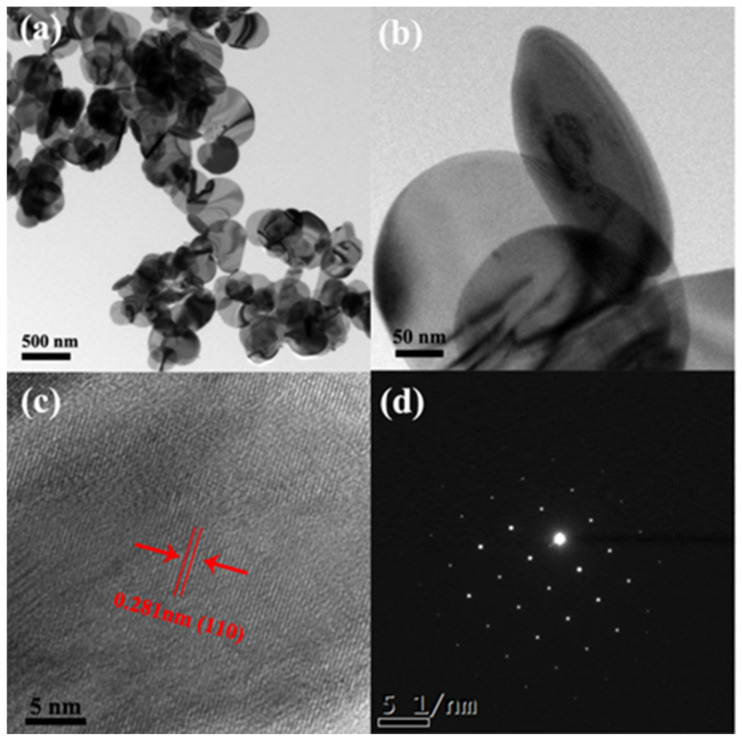
(**a**,**b**) TEM; (**c**) HRTEM images; and (**d**) SAED patterns of BiOBr_0.15_I_0.85_ nanosheets with X = 0.15.

The morphology and textural properties of the as-prepared photocatalysts were studied by scanning electron microscopy (SEM) and high resolution transmission electron microscopy (HRTEM). Figure 2 showed that the morphology of BiOBr_X_I_1−X_ doped with Br in different proportions was nanosheet structure, and the size and thickness changed slightly. It was proven that BiOBr_X_I_1−X_ retained the morphology of nanosheets with changed size and thickness. In Figure 2, the morphology of BiOBr was regular flake and evenly dispersed, which predicts that the synthesis method is reproducible. Replacing the I source with a Br source or Cl source can also prepare BiOBr or BiOCl with flake morphology and high crystallinity. The top-view HRTEM image of the BiOBr_X_I_1−X_ nanosheets with x = 0.15 (Figure 3) revealed highly crystalline and uniform lattice fringes with an interplanar lattice spacing of 0.281 nm indexed as the (110) atomic planes of the BiOBr_0.15_I_0.85_ nanoplate. The corresponding selected area electron diffraction (SAED) pattern (Figure 3d) further proved the single-crystalline feature of the single BiOBr_X_I_1−X_ nanosheets [35,36].

**Figure 4:**
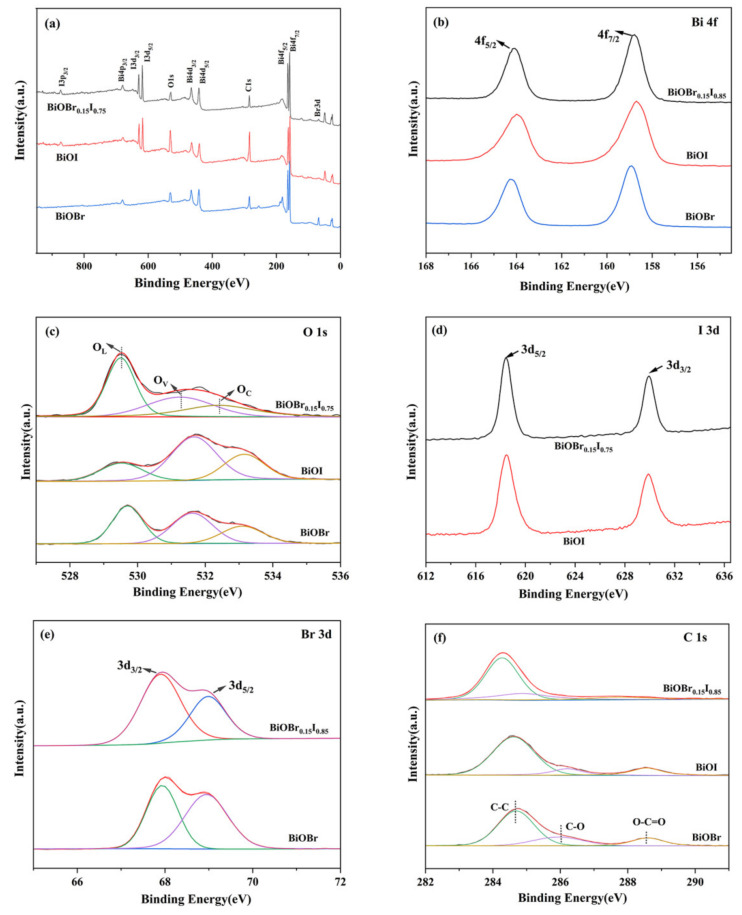
XPS spectra of samples: (**a**) survey; (**b**) Bi 4f spectrum; (**c**) O 1s spectrum; (**d**) I 3d spectrum; (**e**) Br 3d spectrum; and (**f**) C 1s spectrum.

The surface electronic states and chemical compositions of samples were further analyzed by XPS. The survey scan revealed that the surface was mainly composed of Bi, O, I, Br, and a trace amount of C (Figure 4), which indicates the high purity of the BiOBr, BiOI, and BiOBr_0.15_I_0.85_. Compared with the full spectrum of BiOBr and BiOI, (Figure 4a), the orbital peaks of Br 3d appear in the full spectrum of BiOBr_0.15_I_0.75_. The high resolution XPS fine spectra of Bi 4f, O 1s, C 1s, I 3d, or Br 3d were characterized respectively to further analyze the valence changes of various elements in the sample, as shown in Figure 4b–f. As shown in Figure 4b, the peaks at 158.78 eV and 164.08 eV correspond to trivalent Bi 4f7/2 and Bi 4f5/2 orbits, respectively. In Figure 4c, the three main peaks observed at 529.52, 531.28, and 532.43 eV corresponded to the characteristic peak of the Bi-O bond in [Bi_2_O_2_]- layers (OL), oxygen-deficient regions (OV), and hydroxyl groups adhering to the surface (OC), respectively. In Figure 4d, two distinct peaks were located at 618.41 and 629.87 eV, respectively, corresponding to the 3d5/2 and 3d1/2 inner layer electrons of I, indicating that the chemical state of I- in BiOI existed in the form of I- ions. Furthermore, two peaks at 67.83 and 68.93 eV were attributed to Br 3d5/2 and 3d3/2, suggesting that the chemical valence of the Br element was −1 in BiOBr0.15I0.85 [37,38]. In the high resolution C 1s spectrum (Figure 3d), the three sub-peaks respectively correspond to C-C, C-O, and O-C = O. The XPS results supported XRD analysis of the chemical composition of the samples and further confirmed the existence of Br in the BiOI lattice.

**Figure 5:**
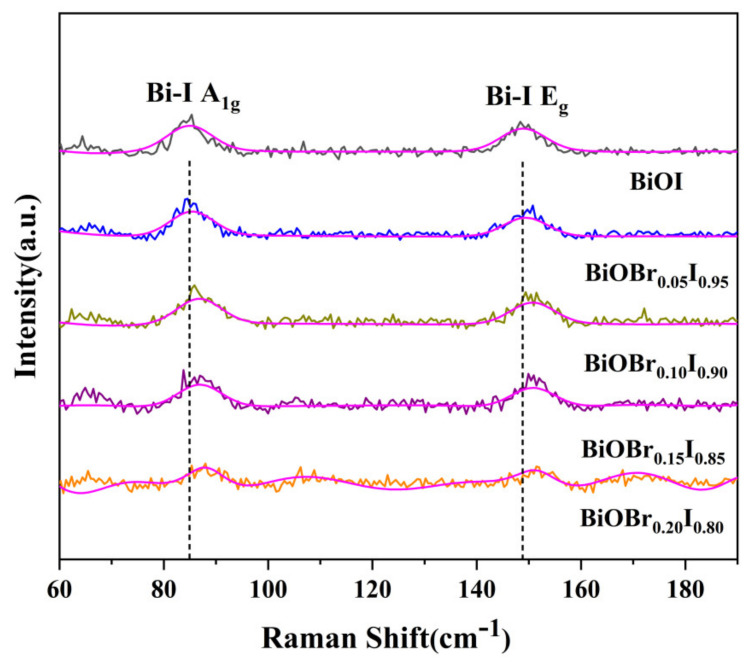
Raman patterns of as-prepared BiOBr_X_I_1−X_ photocatalysts.

To further investigate the chemical bond vibration of the as-prepared samples, Raman spectra of BiOBr_X_I_1−X_ are shown in Figure 5. All samples showed Raman bands of 84.957 and 148.885 cm^−1^, which can be assigned to A1g and Eg of the Bi-I stretching mode, respectively [39]. No other peaks were observed, implying that no other functional groups were formed in BiOBr_X_I_1−X_. The Raman Gaussian fitting information of synthetic samples is summarized in Table 2, including peak position, peak intensity, half height width, etc. With the increase of the Br doping amount, the A1g and Eg Raman peaks of Bi-I bond gradually blue shifted. The reason may be that the lattice distortion caused by doping produces internal stress, accompanied by the decrease of vibration frequency corresponding to Bi-I bond relaxation and the enhancement of vibration scattering. It can be observed that the Raman peak ratio of A1g/Eg in pure BiOI is 1.102. The Br doping process continuously adjusted the intensity of these two kinds of vibration, and the A1g/Eg of BiOBr_0.15_I_0.85_ was the closest to pure BiOI and reached the lowest ratio, 1.148.

**Figure 6:**
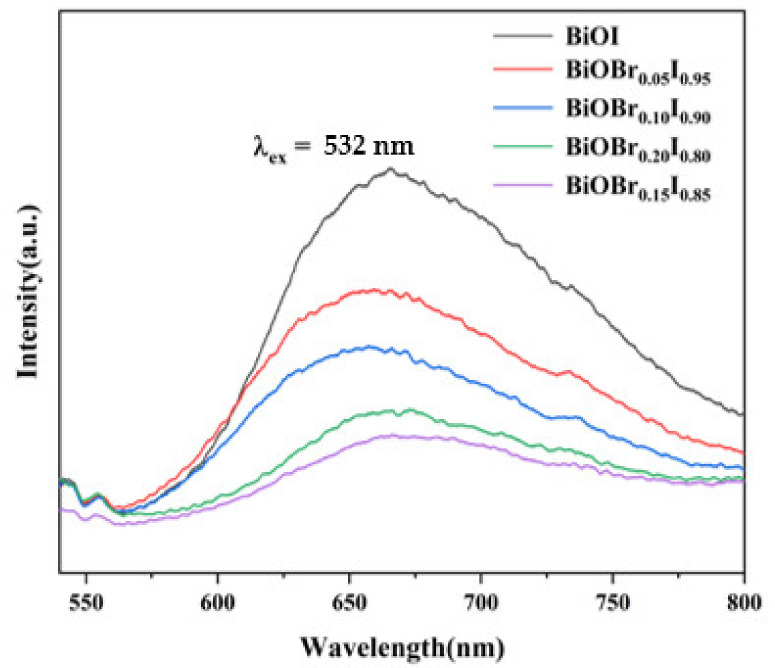
PL spectra of BiOBr_X_I_1−X_ composites and pure BiOI.

The photoelectron-hole separation efficiency of the sample can be investigated by PL emission. Generally speaking, weaker luminescence intensity means less photoelectron-hole recombination and higher photocatalytic activity [40]. As shown in Figure 6, the peak at about 670 nm originated from band-to-band transition of BiOBr_X_I_1−X_ at the excitation wavelength of 532 nm [41]. By forming BiOBr_X_I_1−X_, PL peak intensity of the samples was further decreased, showing that Br replacement doping can restrain the recombination of photo-induced charges. The lower peak intensity of BiOBr_0.15_I_0.85_ compared to other samples suggested a higher separation efficiency of charge carriers, benefiting from the change in energy band position of doped materials.

**Figure 7:**
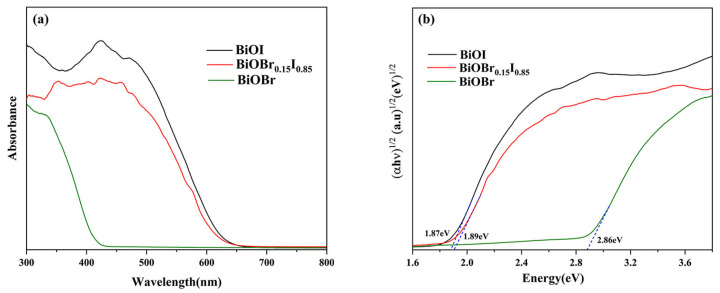
(**a**) UV-vis DRS, and (**b**) the Eg of BiOBr_X_I_1−X_ (x = 0.00, 0.15, 1.00) photocatalysts.

The light absorption capacity of the catalyst had an important effect on the photocatalytic degradation of organic pollutants, so the absorption characteristics were studied by UV-vis DRS. It can be observed in Figure 7a that both BiOI and BiOBr_0.15_I_0.85_ had excellent visible light absorption. The band gap energy of as-prepared samples can also be calculated by fitting a plot of (αhν)^1/2^ versus hν in Figure 7b. The Eg of BiOBr, BiOI, and BiOBr_0.15_I_0.85_ were 2.86 eV,1.87 eV, and 1.89 eV, respectively, which indicates that Br doping clearly changes the absorption properties and widens the bandgap of the solid solution.

**Figure 8:**
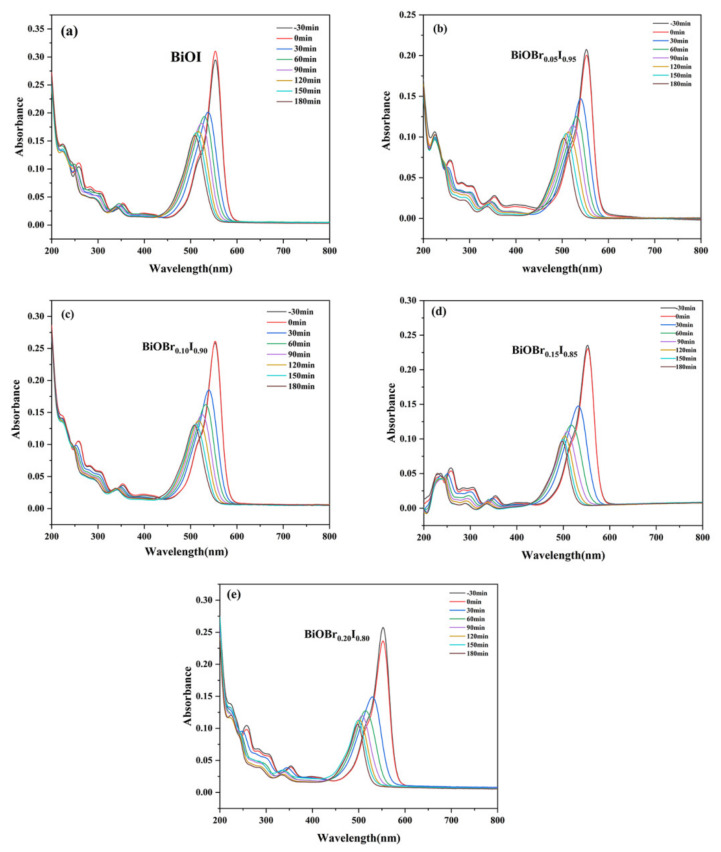
Temporal evolution of the spectra during the photodegradation of RhB mediated by the BiOBr_X_I_1−X_ ((**a**) x = 0.00, (**b**) 0.05, (**c**) 0.10, (**d**) 0.15, (**e**) 0.20) photocatalysts.

The photocatalytic activity of BiOBr_X_I_1−X_ (x = 0.00, 0.05, 0.10, 0.15, 0.20) was evaluated by degrading rhodamine B under Xenon lamp irradiation (λ > 400 nm). Due to the interaction between the electronegative (001) surface exposed by the BiOI photocatalyst and the positively charged cationic dye RhB, the photocatalysts had physical adsorption of RhB within 30 min of dark treatment (Figure 8). It should be noted that the material reached adsorption–desorption dynamic equilibrium (ratio of 1) after dark treatment for 30 min. The degradation rate of RhB was faster under 30 min of light, and then slowed down, accompanied by an obvious “blue shift” of the characteristic peak, indicating that RhB was deethylated to form intermediates.

**Figure 9:**
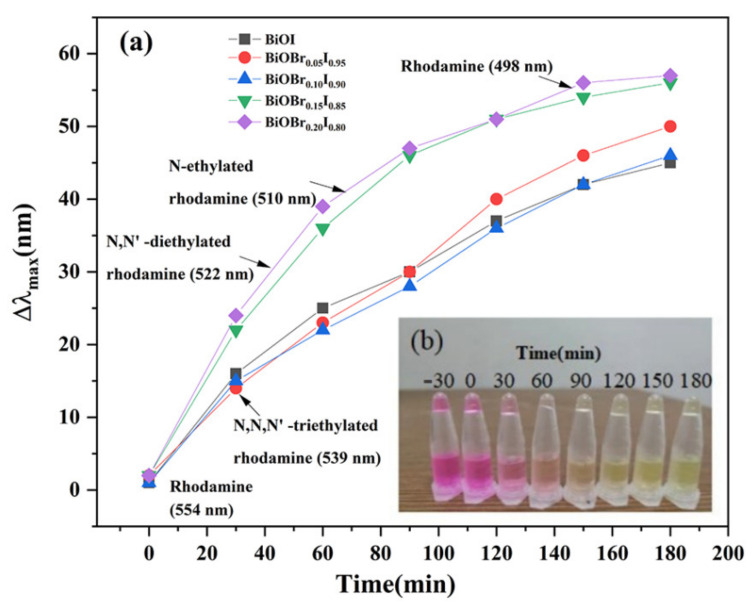
Blue shift in degradation of RhB: (**a**) the “blue shift” of the maximum absorption wavelength of the solution varies with the illumination time; (**b**) the color change of solution during degradation of BiOBr_0.15_I_0.85_.

In order to explore the blue shift phenomenon of peak position and intuitively compare the degree of deethylation, the shift between the characteristic wavelength of the absorption peak and the characteristic wavelength of RhB (554 nm) was analyzed, as shown in Figure 9a. During the photodegradation of RhB, deethylation reactions occurred, including RhB (N,N,N′,N′-tetraethyl rhodamine) at 554 nm, N,N,N′-triethylated rhodamine at 539 nm, N,N′-diethylated rhodamine at 522 nm, N-ethylated rhodamine at 498 nm, and rhodamine at 498 nm [42]. Obviously, the characteristic peak shift did not change after 90 min due to the presence of a small amount of RhB and a large amount of intermediates in the solution, which makes it difficult for the active groups produced in the photocatalytic process to oxidize them. This point can also be confirmed in Figure 9b; the color of the solution started from rose red, gradually changed to light red, and finally turned to yellow, indicating that the number of intermediate products increased and that RhB gradually decreased.

**Figure 10:**
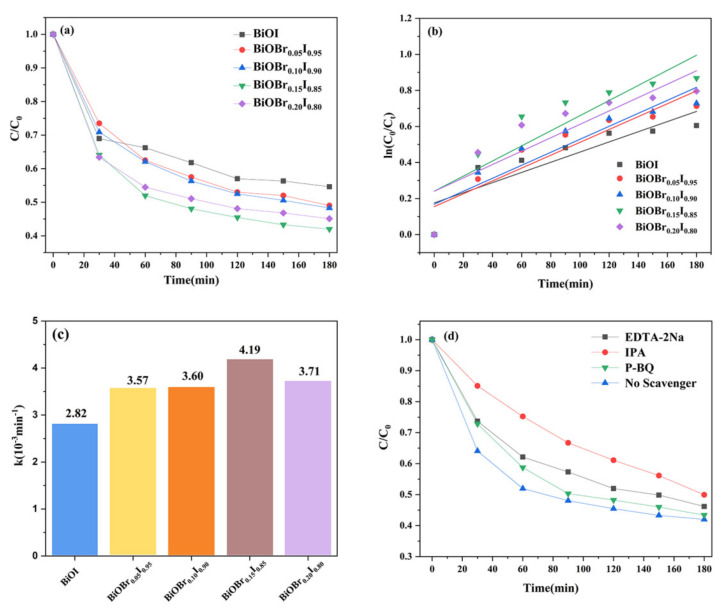
(**a**) Photodegradation of RhB in the presence of different samples; (**b**) kinetic linear simulation curves; (**c**) pseudo-first-order kinetic rate constant k; and (**d**) photocatalytic degradation of RhB over photocatalysts with the addition of P-BQ, EDTA-2Na, IPA, or without scavengers present.

Quantitative photodegradation efficiency and kinetic fitting data are shown in Figure 10a–c. Under visible light irradiation, the degradation rate of BiOBr_X_I_1−X_ (x = 0.00, 0.05, 0.10, 0.15, 0.20) photocatalysts was higher than that of pure BiOI (45.4%), and the photocatalytic degradation rate fell after rising with the increase of Br doping, among which the BiOBr_0.15_I_0.85_ photocatalyst showed the highest degradation rate. It was noteworthy that P25 had a slow degradation rate of 85.81% under the same conditions (Supplementary Figure S1). The doping of Br undoubtedly improved the photocatalytic performance, but the rapid deethylation rate of RhB in degradation led to the production of refractory intermediates. Using the kinetic model, the kinetic fitting curve and kinetic constant histogram can be obtained, as seen in in Figure 10a,b. The reaction rate constants of pure BiOI and BiOBr_X_I_1−X_ (x = 0.00, 0.05, 0.10, 0.15, 0.20) were 2.82, 3.57, 3.60, 4.19, and 3.71 (10^−3^ min^−1^) respectively. The rate constant of BiOBr_0.15_I_0.85_ was 1.49 times that of pure BiOI, indicating that BiOBr_0.15_I_0.85_ had the best photocatalytic performance, which is consistent with the analysis of the degradation curve. The catalytic stability of the catalyst was studied (Figure S2). The results showed that the degradation efficiency of BiOBr_0.15_I_0.85_ for RhB remained stable after three cycles, which proves that it exhibits excellent visible light catalytic stability. The active species capture experiment was carried out to evaluate the active species in photodegradation. In the capture experiment, EDTA-2NA, P-benzoquinone (P-BQ), and isopropanol (IPA) were added to the independent photocatalytic reaction system as hole (H^+^), superoxide radical (·O^2−^), and hydroxyl radical (·OH^−^), respectively [43,44]. When IPA, EDTA-2NA, and P-BQ were added into the reaction system, the degradation rates of the RhB solution decreased from 65.5 to 50.6, 53, and 62.6%, respectively. The results suggested that ·OH^−^ and H^+^ played a leading and secondary role in the photodegradation of RhB, while the influence of ·O^2−^ on the process was almost negligible. The pathway of the photocatalytic reaction was as follows:

BiOI + hv → e^−^ + h^+^ (1)

h^+^ + H_2_O → ·OH + H^+^ (2)

e^−^ + O_2_ → ·O^2−^ (3)

RhB + h^+^/·OH/·O^2−^ → Degradation Products (4)

Due to the close relationship between the positions of the band gap, conduction band, and valence band of catalysts and the photocatalytic process, it was necessary to analyze the change of photocatalytic activity from the perspective of energy band. The valence band and conduction band of a semiconductor at the point of zero charge can be calculated by the following equation:

E_CB_ = λ − E_e_ − 1/2E_g_ (5)

E_VB_ = E_CB_ + E_g_ (6)

where E_VB_ and E_CB_ are the positions of top valence band and bottom conduction band, λ is the absolute electronegativity of the semiconductor, E_e_ is the standard electrode potential of hydrogen (4.5 eV), and E_g_ is the semiconductor band gap value. The conduction band and valence band positions of the sample were calculated from the E_g_ band gap value of diffuse reflection analysis combined with the above formula, as shown in Table 3.

**Figure 11:**
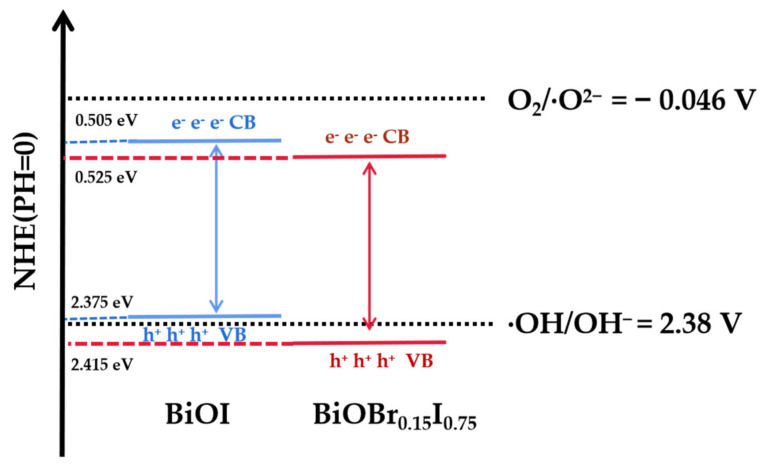
Schematic diagram of the BiOI and BiOBr_0.15_I_0.85_ reaction mechanism for photocatalytic degradation of RhB.

Figure 11 showed the photodegradation mechanism and band structures of BiOBr_0.15_I_0.85_ and BiOI based on the above analysis. The position of the conduction band bottom of pure BiOI was lower than the reduction potential of O^2^/·O^2−^ (0.33 eV) and the position of the valence band top was higher than the oxidation potential of OH^−^/·OH (2.38 eV); only holes (H^+^) and a small amount of hydroxyl radicals (·OH) could be generated in the photocatalytic process, resulting in the poor photocatalytic degradation performance of BiOI [45,46]. Clearly, the widening of the band gaps of BiOBr_0.15_I_0.85_ reduced the absorption of visible light and the downward shift of the valence band position produced more oxidizing holes and hydroxyl radicals, which play an important role in degradation. On the other hand, the extended electron-hole composite path led to more efficient separation of photogenerated carriers in the wider gap of BiOBr_0.15_I_0.85_, consistent with the PL spectral analysis. Therefore, the photocatalytic performance was optimized by optimizing redox potential and blocking carrier recombination.

**Figure 12:**
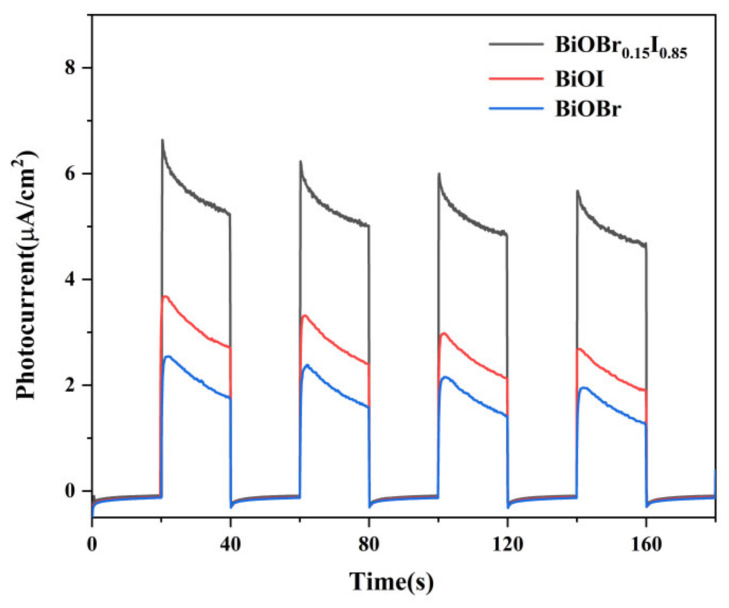
Photocurrent responses of the BiOBr, BiOI, and BiOBr_0.15_I_0.85_ in 0.5 M Na_2_SO_4_ aqueous solutions vs. Ag/AgCl.

To further investigate the photocatalytic mechanism proposed above, the transient photocurrent responses of the BiOBr, BiOI, and BiOBr_0.15_I_0.85_ were measured for several on-off cycles to clarify the interfacial charge separation under intermittent Xe lamp irradiation (Figure 12). It can be clearly seen that all samples showed a stabilized and reversible photocurrent response. The BiOBr_0.15_I_0.85_ exhibited the largest photocurrent density, revealing less recombination and a longer lifetime of photogenerated carriers, which indicates that charge separation efficiency can be enhanced by successfully forming solid solution structure [47]. The enhancement of separation efficiency of photoinduced electron-hole pairs ought to be an important source of excellent photoactivity of band-modulated BiOBr_0.15_I_0.85_ nanosheets, which provides a promising and economical method for the design and development of photodegradation catalysts [48].

## 4. Discussion

In summary, 2D BiOBr_X_I_1−X_ nanoplates were successfully synthesized by a simple hydrothermal method. The photocatalysis performance of those as-prepared samples was evaluated by RhB degradation under visible light. The variation of composition of BiOBr_X_I_1−X_ solid solutions led to changes of its optical and structural characteristics, which significantly affected the photocatalytic activity. Further, the possible photocatalytic mechanism was clarified according to the free radical capture experiment. It is worth noting that the BiOBr_0.15_I_0.85_ nanoplate with excellent electron hole separation efficiency possessed the best photocatalytic activity. The formation of solid solution successfully adjusts the transport path of effective carriers and enhances the oxidizability of free radicals, which provides a good path for future treatment of environmental and energy problems.

## Figures and Tables

**Table 1:** Lattice constant of BiOBr_X_I_1−X_.

Sample	x = 0.00	x = 0.05	x = 0.10	x = 0.15	x = 0.20
a (Å)	3.996	3.993	3.989	3.985	3.979
b (Å)	3.996	3.993	3.989	3.985	3.979
c (Å)	9.155	9.152	9.126	9.103	9.069
v (Å^3^)	146.173	145.916	145.204	144.586	143.594
(β,γ)	90.000	90.000	90.000	90.000	90.000
R_wp_ (%)	8.67	9.51	9.14	9.14	9.00
R_p_ (%)	6.32	7.02	6.79	6.80	6.78

**Table 2:** Raman fitting of BiOBr_X_I_1−X_.

Sample		x = 0.00	x = 0.05	x = 0.10	x = 0.15	x = 0.20
Bi-I (A_1g_)	Raman shift	84.957	85.388	86.771	86.869	87.968
	Peak strength	35.781	36.520	36.797	34.290	49.012
	Half height width	9.882	9.882	9.882	9.882	9.882
	Peak area	376.363	384.139	387.048	360.676	615.654
Bi-I (E_g_)	Raman shift	148.885	149.384	150.669	150.824	151.452
	Peak strength	32.477	28.135	31.334	29.870	34.955
	Half height width	9.882	9.882	9.882	9.882	9.882
	Peak area	341.605	295.940	329.589	314.190	442.096
A_1g_/E_g_	Peak ratio	1.102	1.298	1.174	1.148	1.402
	Peak area ratio	1.102	1.298	1.174	1.148	1.393

**Table 3:** Band structure parameters of samples (unit: eV).

Sample	E_g_	E_V_	E_C_
BiOI	1.87	2.375	0.505
BiOBr_0.15_I_0.85_	1.89	2.415	0.525
BiOBr	2.86	3.100	0.240

## Data Availability

Not applicable.

## Supplementary Materials

The following are available online at https://www.mdpi.com/article/10.3390/nano11112940/s1, Figure S1: Photodegradation rate of P25 to RhB; Figure S2: Cycling experiments on RhB photodegradation over BiOBr0.15I0.85; Table S1: Comparison of degradation efficiency of related photocatalysts.
