# Supplementary material for: Synthesis and Enhanced Light Photocatalytic Activity of Modulating Band BiOBrXI1−X Nanosheets
Source: Nanomaterials (Basel). 2021 Nov 2;11(11):2940. doi: 10.3390/nano11112940 (PMC8619692; doi:10.3390/nano11112940)
Supplement: Supplementary file 1 [file nanomaterials-11-02940-s001.zip › nanomaterials-1417465-supplementary.pdf]

nanomaterials

Supporting Informationp

Synthesis and Enhanced Light Photocatalytic Activity of Modulating Band BiOBrxI1–x Nanosheets

Bingke Zhang [1,2,†], Shengwen Fu [1,2,†], Dongbo Wang [1,2,*], Shujie Jiao [1,2,*], Zhi Zeng [1,2], Xiangyu Zhang [1,2], Zhikun Xu [3,*], Yaxin Liu [1,2], Chenchen Zhao [1,2], Jingwen Pan [1,2], Donghao Liu [1,2] and Jinzhong Wang [1,2,*]

[1] National Key Laboratory for Precision Hot Processing of Metals, Harbin Institute of Technology, Harbin 150001, China; zhangbingke007@163.com (B.Z.); wangdong165@sina.com (S.F.); zengzhi@hit.edu.cn (Z.Z.); zhangxiangyu@163.com (X.Z.); 20S009004@stu.hit.edu.cn (Y.L.); chenzhao@hit.edu.cn (C.Z.); Panjw19@163.com (J.P.); 19S009009@stu.hit.edu.cn (D.L.)
[2] Department of Optoelectronic Information Science, School of Materials Science and Engineering, Harbin Institute of Technology, Harbin 150001, China
[3] College of Science, Guangdong University of Petrochemical Technology, Guandu Road No. 139, Maoming 525000, China
* Correspondence: wangdongbo@hit.edu.cn (D.W.); shujiejiao@hit.edu.cn (S.J.); xuzhikunnano@163.com (Z.X.); jinzhong_wang@hit.edu.cn (J.W.)
† Both authors contributed to this work equally.

Citation: Zhang, B.; Fu, S.; Wang, D.; Jiao, S.; Zeng, Z.; Zhang, X.; Xu, Z.; Liu, Y.; Zhao, C.; Pan, J.; et al. Synthesis and Enhanced Light Photocatalytic Activity of Modulating Band BiOBrxI1-x Nanosheets. *Nanomaterials* **2021**, *11*, 2940. https://doi.org/10.3390/nano11112940

Academic Editor: Vincenzo Vaiano

Received: 27 September 2021
Accepted: 28 October 2021
Published: 2 November 2021

Publisher's Note: MDPI stays neutral with regard to jurisdictional claims in published maps and institutional affiliations.

Copyright: © 2021 by the authors. Licensee MDPI, Basel, Switzerland. This article is an open access article distributed under the terms and conditions of the Creative Commons Attribution (CC BY) license (http://creativecommons.org/licenses/by/4.0/).

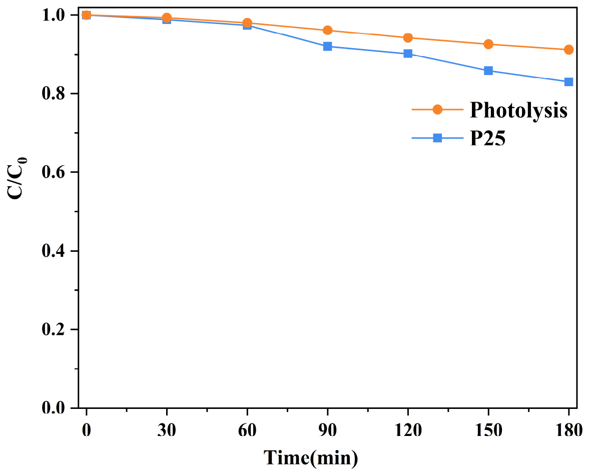

Figure S1. Photocatalytic degradation of RhB in the presence of P25 and photolysis of RhB under visible light irradiation.

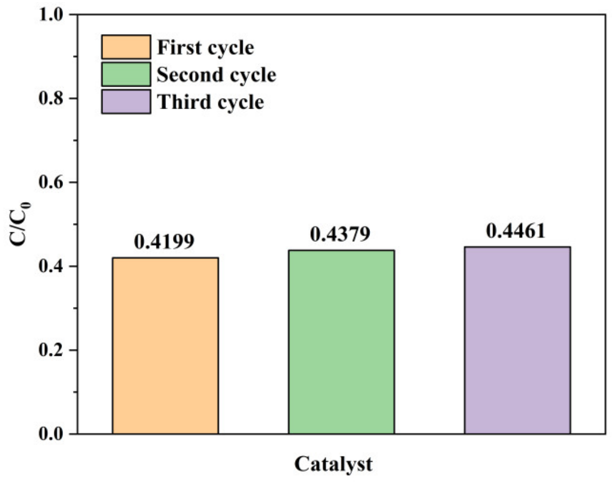

Figure S2. Cycling experiments on RhB photodegradation over $BiOBr_{0.15}I_{0.85}$.

Table S1. Comparison of degradation efficiency of related photocatalysts.

Catalyst.	Pollutant	Catalyst amount (g/L)	Light source	Time (min)	Degradation (%)	Ref.
g-C_3N_4/BiOl/BiOBr	Mo	5.0	Visible light	300 min	83.7%	[1]
BiOI nanoplate	Mo	0.3	Visible light	180 min	~21%	[2]
BiOI@rGO	RhB	1.0	Visible light	180 min	~85.0%	[3]
BiOI@$Bi_{12}O_{17}Cl_2$	RhB	1.0	Visible light	5 h	~91.0%	[4]
Ag/BiOI	RhB	1.0	Visible light	4 h	~49.6%	[5]
BiOI	RhB	0.1	visible light (white LED)	12 h	~70%	[6]
$BiOIO_3$/BiOI	RhB	0.5	UV-visible light	90 min	~98%	[7]
$CoFe_2O_4$ /BiOBr	RhB	0.25	Visible light	6 h	~95%	[8]
BiOClBr	RhB	0.3	420nm	8 h	~49.8%	[9]
$BiOBr_{1-X}I_X$	RhB	0.5	Visible light	180 min	~58.0%	This work

References

1. Liu, B.; Han, X.; Wang, Y.; Fan, X.; Wang, Z.; Zhang, J.; Shi, H. Synthesis of g-C3N4/BiOI/BiOBr heterostructures for efficient visible-light-induced photocatalytic and antibacterial activity. *J. Mater. Sci.: Mater. Electron.* **2018**, *29*, 14300–14310, https://doi.org/10.1007/s10854-018-9564-4.
2. Xia, J.; Yin, S.; Li, H.; Xu, H.; Yan, Y.; Zhang, Q. Self-Assembly and Enhanced Photocatalytic Properties of BiOI Hollow Microspheres via a Reactable Ionic Liquid. *Langmuir* **2010**, *27*, 1200–1206, https://doi.org/10.1021/la104054r.
3. Wang, H.; Liang, Y.; Liu, L.; Hu, J.; Wu, P.; Cui, W. Enriched photoelectrocatalytic degradation and photoelectric performance of BiOI photoelectrode by coupling rGO. *Appl. Catal. B: Environ.* **2017**, *208*, 22–34, https://doi.org/10.1016/j.apcatb.2017.02.055.
4. Huang, H.; Xiao, K.; He, Y.; Zhang, T.; Dong, F.; Du, X.; Zhang, Y. In situ assembly of BiOI@Bi12O17Cl2 p-n junction: charge induced unique front-lateral surfaces coupling heterostructure with high exposure of BiOI {001} active facets for robust and nonselective photocatalysis. *Applied Catalysis B: Environmental* **2016**, *199*, 75–86, https://doi.org/10.1016/j.apcatb.2016.06.020.
5. Liu, H.; Cao, W.; Su, Y.; Wang, Y.; Wang, X. Synthesis, characterization and photocatalytic performance of novel visible-light-induced Ag/BiOI. *Appl. Catal. B: Environ.* **2012**, *111-112*, 271–279, https://doi.org/10.1016/j.apcatb.2011.10.008.
6. Florez-Rios, J.F.; Santana-Aranda, M.A.; Quiñones-Galván, J.G.; Escobedo-Morales, A.; Chávez-Chávez, A.; Pérez-Centeno, A. Alternative Bi precursor effects on the structural, optical, morphological and photocatalytic properties of BiOI nanostructures. *Mater. Res. Express* **2020**, *7*, https://doi.org/10.1088/2053-1591/ab677b.
7. Lu, M.; Xiao, X.; Zeng, G. Bi2S3 nanorods and BiOI nanosheets co-modified BiOIO3 nanosheets: an efficient vis-light response photocatalysts for RhB degradation. *J. Alloy. Compd.* **2021**, *885*, 160996, https://doi.org/10.1016/j.jallcom.2021.160996.
8. Choi, Y.I.; Kim, Y.-I.; Cho, D.W.; Kang, J.-S.; Leung, K.T.; Sohn, Y. Recyclable magnetic CoFe2O4/BiOX (X = Cl, Br and I) microflowers for photocatalytic treatment of water contaminated with methyl orange, rhodamine B, methylene blue, and a mixed dye. *RSC Adv.* **2015**, *5*, 79624–79634, https://doi.org/10.1039/c5ra17616f.
9. Mao, X.-M.; Fan, C.-M. Effect of light response on the photocatalytic activity of $BiOCl_xBr_{1-x}$ in the removal of Rhodamine B from water. *Int. J. Miner. Metall. Mater* **2013**, *20*, 1089, https://doi.org/10.1007/s12613-013-0838-9.
